# Planning For Unmet Social Needs In Pandemics: Teachers’ Experiences Of Unacknowledged Care Work

**DOI:** 10.1093/phe/phag019

**Published:** 2026-06-09

**Authors:** Jane Williams, Claire Hooker, Gwendolyn L Gilbert, Suyin Hor, Laura Donovan, Chris Degeling

**Affiliations:** Sydney School of Public Health, The University of Sydney, Camperdown, NSW 2006, Australia; Sydney School of Public Health, The University of Sydney, Camperdown, NSW 2006, Australia; Sydney School of Public Health, The University of Sydney, Camperdown, NSW 2006, Australia; School of Public Health, University of Technology Sydney, NSW 2007, Australia; ACHEEV, School of Social Sciences, University of Wollongong, NSW 2522, Australia; ACHEEV, School of Social Sciences, University of Wollongong, NSW 2522, Australia

## Abstract

This paper examines the experiences of teachers in Australia during the COVID-19 pandemic from a care ethics perspective. While pandemic preparedness plans prioritised continuity of services and infection control, they failed to anticipate the relational and moral labour required of frontline workers in non-clinical roles. Paradoxically, otherwise prudent and precautionary responses to the risks posed by the pandemic established new barriers to care and human connection and exacerbated existing ones. Drawing on 32 qualitative interviews with teachers, we analyse how they engaged in improvised, context-specific practices of care. We distinguish between care initiated by workers and care requested of them, highlighting asymmetries. We argue that the pandemic exposed and intensified structurally patterned inequities in the distribution and valuation of care work and that existing accounts of care ethics do not sufficiently explain community care performed by teachers. The paper calls for pandemic planners to explicitly anticipate the needs that public health restrictions create and acknowledge and centre care as a communal good. This is because when care is invisible, it is not planned for, acknowledged or supported. Ignoring it exposes persistent institutional failures to recognise and sustain the everyday labour that holds society together in times of crisis.

## Background

### Essential Work and COVID-19

At the beginning of the COVID-19 pandemic, care scholars Michael Fine and Joan Tronto called attention to the ‘less visible care workers who are often ignored or forgotten’ (Fine and Tronto, 2020). These workers were certainly seldom visible in the pandemic preparedness plans that many governments, including Australia’s, had put in place prior to the COVID-19 pandemic (World Health Organisation, 2011; Australian Government Department of Health, 2019). Those plans were largely predicated on the anticipated emergence of a novel influenza A virus strain with semi-predictable rate of spread, mortality and morbidity. Accordingly, they focused on health systems responses. Where other organisations had preparedness plans, these were focused on business continuity to ensure economic viability and continuation of essential services for customers. As Fine and Tronto indicated, there was little consideration of workers’ welfare.

As is well recognised, the novel SARS-CoV-2 pandemic was very different from an expected influenza pandemic, exposing the limitations of existing pandemic preparedness plans. SARS-CoV-2 was more novel, uncertain and, for a long time, threatening. COVID-19 placed unprecedented strain or weight on many long-standing and often taken for granted social institutions with major disruptions to education, employment, and public and private life. Somewhat paradoxically, otherwise prudent and precautionary responses to the risks posed by the pandemic established new barriers to care and human connection and exacerbated existing ones. Existing care infrastructures had to be stretched, reconfigured or made newly visible in the context of systemic disruption but, as Fine and Tronto predicted, this continued to command comparatively little attention. Yet these infrastructures were those that primarily shaped the experience of the pandemic for most members of the public. Six years on, governments around the world are releasing the findings of reviews that are intended to inform preparations for future pandemics.

Fine and Tronto asked particularly what we can learn about those workers whose labour constructed and enacted these infrastructures, that is, what we can learn from care workers’ experiences of essential work during the pandemic (Fine and Tronto, 2020). This is critically important, because who is deemed ‘essential’ and what constitutes ‘essential work’ in times of crisis are not fixed categories but socially constructed and context dependent. As the pandemic began, ‘essential work’ was largely synonymous with healthcare, in public discourse at least. However, as it progressed, the term expanded to include workers who kept food processed, shelves stocked, transport systems running, children educated and the elderly supported, alongside first responders and major trades. The focus, however, was on keeping services functioning rather than the welfare of essential workers.

The pandemic did not simply test institutions; it revealed the relational fragility and structural dependencies that sustain everyday life in late modern societies. Furthermore, the pandemic did not merely reveal vulnerability—it revealed the politics of vulnerability (The Lancet, 2020). Feminist scholars have argued that care is not a private virtue alone but a public, political necessity, one that underpins societal functioning yet remains consistently undervalued, racialised, feminised and precariously organised (Tronto, 1993; Noddings, 2013). Tronto insists that to care well, societies must attend not only to immediate needs, but also to the structures of power and injustice that determine who bears the burden of care, under what conditions and with what recognition or support (Tronto, 1993). As the often-invisible webs of care that underpin social life were strained, it became clear that the capacity to care—and the likelihood of being cared for—was unevenly distributed across socioeconomic, occupational and gendered lines.

### Teaching and an Ethics of Care

What constitutes care is not fixed. The ethics of care literature variously encompasses paid work, attachment and virtue (Gheaus, 2022). For the purposes of this paper, we focus on activities that were relayed to us as care within the broad confines of employment. Paid care, in the literature, tends to focus on those roles that include physical care—aged care, disability care and healthcare—rather than education providers. Foundational ethics of care literature that pertains to teaching leans on virtue or on modelling how to care in the context of moral education (Noddings, 1996, 2013). More broadly, the classroom setting and physical proximity are central to accounts of care and the ethics of care in teaching (e.g. Vogt, 2002; Noddings, 2017; Jackson, 2025). Work that focuses on care ethics and online education is post-secondary and pedagogically focused and is not contextualised in exceptional and compulsory remote schooling (e.g. Rabin, 2021). Common to theories of care is its relational and reciprocal nature, the shifting roles of what Noddings terms the ‘one-caring’ and the ‘cared for’. These roles may be asymmetric and involve power imbalances, but, according to Mol, the shifting practices of care mean that ‘one moment you care and the next you are taken care of’ (Mol, 2008).

### Tinkering and Care

In the introduction to their edited collection of essays on *Care in Practice*, Annemarie Mol, Ingunn Moser and Jeannette Pols describe care ethics in practice as ‘persistent tinkering in a world full of complex ambivalence and shifting tensions’, where care ‘seeks to lighten what is heavy’ (Mol *et al.*, 2010) (p14). ‘Tinkering’ has its origins in constructivism, highlighting the messy realities and workarounds that are obscured from many accounts of the making of science.(Knorr, 1979) Since then, it has been employed in the realm of care to emphasise the relational and fluid nature of care, contrasting care practices with a strict adherence to the standardisation and rules that often govern the administration of care (Mol, 2008). Tinkering is also usefully employed to explain how humans interact or interfere with technologies that are not quite fit for service (e.g. Pols and Willems, 2011; Meyer *et al.*, 2025). In the context of aged care, Molterer *et al*. helpfully combine the relational with the professional while acknowledging that each attends to different intended goods and claim that it is the context-based tinkering processes between those goods that lead to a practical ethics of care (Molterer *et al.*, 2020). We use the idea of tinkering—making small changes, fiddling with detail in order to make something work—to consider how teachers enacted unusual care during the COVID-19 pandemic. They did so not as a moral ideal but as a practical, situated and frequently unacknowledged response to rapidly shifting social needs when the regular parameters of their jobs constrained it.

### Pandemic Planning

The roles of teachers and schools were largely absent from pandemic planning, yet they were essential when a pandemic unfolded. Public health restrictions required teachers to adapt, improvise and continue working, under volatile conditions, and spotlighted by public discourse. Teachers faced challenges as a result of the circumstance of becoming unexpected frontline personnel in a pandemic. As a large body of international literature attests, they experienced burnout and significant stress while adapting to online learning (Beames *et al.*, 2021; Pressley, 2021; Shimony *et al.*, 2022). Remote schooling exacerbated existing inequalities among students (Dimopoulos *et al.*, 2021), often led to poorer educational outcomes (König and Frey, 2022) and negatively impacted the wellbeing of teachers, students and their parents/carers (Connor *et al.*, 2022). In Australia, the COVID-19 pandemic further exacerbated existing struggles in the teaching profession and precipitated attrition (Van Bergen and Daniel, 2023).

This study explores how forms of care were enacted through situated responses to institutional breakdown and how workers navigated moral ambivalence within a deeply political landscape of care. We examine the out-of-the-ordinary care undertaken by teachers during periods of the COVID-19 pandemic when students were learning remotely and the tinkering with professional norms and pandemic requirements that were needed to make it happen. We ask what their experiences can contribute to a growing literature on improved and equitable pandemic preparedness.

## Methods

This paper reports on a qualitative interview study of teachers (*n* = 32) carried out in New South Wales (NSW) and Victoria, Australia. The interviews formed part of a larger programme of research about social preparedness for future infectious disease emergencies with a particular focus on essential workers and were designed to capture the experiences of teachers during periods of lockdown or ‘stay at home orders’ in the first 2 years of the pandemic.

We used multiple approaches to recruit participants, including news media, fliers on noticeboards, social media advertisements and a professional research services provider. Recruitment was initially restricted to NSW, where we focused on recruiting participants in a mix of year levels, school types and locations. After we had completed 25 interviews, we purposively sampled teachers in Melbourne, Victoria (*n* = 7), to try to understand how the longer, more intensive restrictions in that state had shaped teachers’ experiences. Participants chose the mode (telephone or online video conferencing) and time for interview. Verbal consent was obtained and recorded prior to each interview. Participants were compensated for their time with a supermarket voucher. Ethics approval was granted by the University of Wollongong Human Research Ethics Committee.

Data collection began in June 2022 and ended in May 2023. The author team jointly developed the initial interview guide based on previous work on pandemic planning, COVID-19 response and education. JW and CD took a collaborative approach to data collection and analysis. We began data collection by each separately conducting five interviews and meeting after each interview to debrief. We did this to refine the interview guide to be appropriately responsive to both our research questions and the participants’ experiences. JW conducted the remaining 22 interviews. Questions focused both on practical matters and on themes such as respect, trust, uncertainty and stigma. Interviews lasted between 25 and 63 minutes, with a median time of 43 minutes. Interviews were audio recorded and professionally transcribed. Transcripts were de-identified and participants were given pseudonyms.

Analysis was also initially collaborative. JW and CD separately conducted high level inductive coding of the first 10 transcripts as data were collected. As we met after each interview, we began to develop codes to explore the themes that we were identifying, guided by the principles and procedures of reflexive thematic analysis (Braun and Clarke, 2019). We used rolling memos to note ideas, establish and build on themes and reflect on silences and unexpected responses. JW continued to memo and coded all interviews in nVivo. Codes focused on practical place and space-related changes and adaptations that teachers made to respond to the pandemic and the emotional burdens associated with interactions with students, families, principals and the Department of Education. We did not verify our analyses with participants but did feed results back in the form of a lay summary.

We each brought different areas of interest and focus to data collection and analysis. Combining and comparing insights lent richness to our understanding of participants’ experiences. We inevitably also brought our own experiences of COVID-19 restrictions to our interviews and analysis; JW and CD were living and working in different locations under COVID-19 rules of different intensity, both had children learning at home for prolonged periods and at different levels of schooling and different types of school. The wider authorship team met every 4 weeks from the beginning of the project. We discussed and explored findings, identified and drew on links with other studies and reviews and considered the resonance of our own analysis.

### Participant Data

Participants (*n* = 32) were employed at 36 different schools and had a wide range of ages and levels of experience. Some had worked in more than one school during 2020–2021 because of a change in employment. They worked at schools in the public (state-funded) (*n* = 21), independent (private, including non-Catholic religious) (*n* = 8) and Catholic (*n* = 7) education systems, in metropolitan (*n* = 14), regional (*n* = 9) and rural (*n* = 2) settings in NSW and metropolitan Victoria (*n* = 7). More participants worked at secondary (grades 7–12) (*n* = 25) than primary (kindergarten–grade 6) (*n* = 7) schools. There were more female (*n* = 21) than male (*n* = 11) teachers, but their participation was proportionate to employment ratios in the education workforce.

## Results

Participants discussed the many instances in which they engaged in care practices that exceeded their formal job descriptions and the novel ways they worked to enact them. Practices included both (i) initiating care that fell outside of their core work functions and (ii) having regular care requests made of them that they felt unqualified or unwilling to provide, but invariably responded to anyway. Care *initiated by* teachers was defined as care they considered as being within their scope of work and which they offered in response to need. For example, teachers provided additional emotional support for students that they felt was needed in the context of extended remote learning. Care *requested of* workers was that which they felt obligated to provide because it was asked of them. Whether initiated by or requested of participants, the new care activities required the workers to ‘tinker’ with the parameters of their normal roles and the COVID-19 systems and barriers in place. Such tinkering was often rooted in physical restrictions and workarounds.

As teachers discussed their experiences of the unusual requirements of their jobs during periods of remote learning, it was striking how little of what they relayed was related to educational goods. Some expressed concern about the impact of remote schooling on students’ learning and noted ongoing challenges associated with learning disruption. However, the significant bulk of their reported experiences related to those other goods that are not the core business of schools but nevertheless can be provided in the school setting: safety, food, resources, counsel and social connection. It was those missing social goods that teachers sought to support with their efforts.

### Caring Outside the Classroom

Teachers in all locations and school types reported caring for families, in addition to the students in their classes. They counselled parents over the phone. They dropped care packages and food for students and their siblings and parents. Some teachers reported going to students’ homes and standing on the doorstep or over the fence, so they could engage with the student and their family in person.

Zach, a high school teacher in Sydney’s southwestern suburbs, recalled:

As we went on, we noticed that a lot of the families, especially for our lower socioeconomic kids, we started doing food drives and things like that because we realised that education was the least of their concerns. A lot of [the parents] weren’t working and they didn’t have an income. And we were then having to organise with the outside agencies, okay, we have to start delivering food.

His experience was typical of teachers working in locations that were more severely impacted by COVID-19 job losses, and many of the teachers we interviewed bought and/or delivered food and care packages and educational equipment to students. Most said they paid for food and small extras out of pocket but some said they advocated on families’ behalf with external organisations that were equipped to provide more care than the teachers could handle. Many arranged access to electronic devices or internet access at students’ homes.

Janine, a special education teacher in Melbourne, described a different kind of delivery:

We delivered a care package to every student. As teachers, we were okay. It had things like some tissues. It had some hand sanitiser. It had some sudokus and colouring and pencils to do those little adult colouring and that sort of thing. It had a chocolate bar. It had something for the mind, something to do. So, maybe a little craft-type thing to do. It was really a ‘We’re thinking of you’… And that was very well received by students particularly because these kids, well, you couldn’t go out and buy things, but also, they didn’t have the money to be able to buy things like that. And it was something just for them.

In contrast to Zach, Janine’s deliveries were less about practical essentials like food and more about the need for social connection. She attended to essential needs that are undervalued and therefore not prioritised by governments and other institutions.

Care that involved visiting students and their families was also an attempt to assuage teachers’ worries. They worried about students who were vulnerable and at risk and about families that were struggling. Worry was compounded by not being able to see their students. This concern was particularly reported by teachers who worked in low-income settings, in schools that educate students with additional needs or that serve tight-knit communities (usually religious or ethnic minorities or geographically isolated). Participants worried about violence and abuse in students’ homes, described by Rose as an ‘extra mental burden that you really can’t quantify’. Distance learning was, for some teachers, inadequate because, as Zahra said, ‘If a child wasn’t online, how do we know that they’re safe, how do we know that they’re alive?’. Participants were reassured by seeing a student, at least for a time. This type of care (visits and care packages) was initiated by, rather than requested of, participants, who did not describe it as burdensome. However, the worry that prompted it was.

Attending school in person while stay at home orders were in place in 2020–2021 was officially discouraged for children who had supervision at home. The care that teachers reported providing during the COVID-19 pandemic was therefore different from that expected of them when teaching outside of pandemic restrictions. Ordinarily, hungry children can be fed at school, formally or informally. The wellbeing and safety of children with difficult home lives can be monitored at school. Spatial distancing restrictions meant that teachers tinkered with usual practice to act in ways that would usually be unnecessary and outside the scope of their usual jobs. They delivered goods, stood on doorsteps or had conversations over fences or through windows. They arranged for the provision of technological goods in homes that were, for some students, usually the sole preserve of the classroom. The care that teachers were accustomed to providing within the scope of their roles could not be enacted in usual ways, so they took new actions to make it possible. Some acknowledged the limitations of the care that could be provided in this way and encouraged ineligible students to attend school because of safety concerns at home.

### The Obligation to Care

Motivations for providing care varied. While participants described scenarios where they willingly tinkered with systems to provide care, some also reported feeling resentful as bending normal practices made teachers more vulnerable to others’ needs. Many teachers objected to having to provide mental healthcare to students and their extended families, for which most felt unqualified. Although most described this type of care as time-consuming and burdensome, teachers in tight-knit communities were less likely to express it as an unwanted load. Sophie, a primary school teacher in Sydney, describes a typical scenario:

So, we’d have a lot of really long phone calls with parents, who were anxious themselves. And at times it felt like we were not only being counsellors and teachers to the students, but we were also being counsellors to the parents. Partly to assuage their guilt, to the fact that they have to work and can’t concentrate on their child’s learning at home, or to reassure them that their child is going to be okay. And again, that’s very much beyond the scope of what we’re trained to do. We just do it, but it’s certainly not within our job description.

Or, as Rose, a high school teacher in Melbourne, put it ‘you were expected to be social workers and counsellors before you’re even teachers’. Although many mentioned that they felt unqualified to provide the psychological support they were expected to give, none reported withholding it. Altering the norms of classroom connection made it easier for families to overlook the normal boundaries between teachers and students. Teachers continued to provide requested care that fell outside of what was usual, non-pandemic, practice but felt that being more available came at some expense.

### Patterns of Care and Community Duty

Not all participants reported a connection with community, and a few said that the only difference in their role was having to shift online. These participants were more likely to be younger and less experienced high school teachers located in capital cities who did not live in the community they worked in. Teachers who reported initiating care for students’ wider families tended to work in close knit communities of varying descriptions. Some worked in geographically isolated areas where people were more reliant on neighbours and community members, some in low socioeconomic areas heavily impacted by pandemic restrictions and others at religious schools that were the heart of a cultural community. In these settings, teachers were more likely to be known in the community and to know the siblings and wider families of the students in their classes, or at least to be familiar with their lives. This embeddedness made them more likely to initiate care beyond their professional remit.

### Caring and Being Cared For

Almost all the participants in this study talked about asymmetries of care, of providing care while not being or feeling cared for themselves. Many teachers reported unreasonable demands on their time to accommodate the additional work required of them by COVID-19 restrictions and said they felt angry and resentful. This was usually time that was required to accommodate altered systems, such as checking in individually with each child outside of class time and marking submitted work (rather than being able to look over a student’s shoulder) or alerting parents daily to their child’s non-participation.

We described above the flexibility that some teachers voluntarily exhibited in going to their students when they felt it was warranted. Work outside of normal work environments and normal work hours was also reportedly required of participants. Many reported receiving calls and emails from students and families around the clock, and one teacher was required to work from her car, parked outside her home. Siobhan reported:

The principal was very big on […] we had to have a professional background, we had to have no interruptions. Our family members couldn’t come and interrupt us. We couldn’t have noise in the background. We had to still have that professional teacher online presence. So I ended up doing my Google Meets from my car just because my own children would get off their Google Meets and then they would be noisy as children are. We weren’t allowed to have anyone interrupting our Google Meets. So I had to do mine in my car where it was totally quiet and totally silent and I wouldn’t be interrupted. So I’m sitting out there in my car doing Google Meets thinking, oh my goodness, what is this? And I had parents commenting, ‘Oh, are you sitting in your car?’ It’s like, yeah, well this is the only quiet place I’ve got.

A more common scenario was a reported asymmetry of care. Some employers gave teachers care packages as an acknowledgment of the additional burdens they were carrying. Ironically, these were frequently perceived as inadequate and, in some cases, exacerbated feelings of resentment among staff. Teachers viewed such efforts as a misalignment of responsibility: when they themselves provided care packages to students and their families it was an expression of care that lay outside the formal remit of their roles. They felt, however, that a manageable workload and supportive work environment was a core obligation of employers that had not been fulfilled and that ‘care packages’ were a poor substitute. Their own needs were unmet. Participants frequently described feeling disrespected by more remote power holders, such as politicians, who ‘threw teachers under the bus’, and the Department of Education who, participants felt, had created unrealistic expectations of teachers and teaching during the pandemic.

One exceptional scenario was a small number of teachers who reported feeling cared for by their students. This cohort of teachers was older and less technologically adept, but highly experienced with and confident in their classroom teaching roles. They reported initial difficulty functioning in the online space that was suddenly required of them and said their students tried hard to help them, troubleshooting tech issues and sending recommendations for technological workarounds to make teaching better. This helped those teachers function more effectively in the online space that was suddenly required of them and they described the help they received from students as care.

Participants made consistent calls for improvement; all involved being, or at least feeling, better valued. Many remarked that the blurring of school and home boundaries that had occurred during lockdown had persisted past periods of COVID-19 emergency restrictions and now constituted a ‘new normal’. Angela describes the shift:

Because parents – and unfortunately, I’ve noticed this after the pandemic, they still expect us to be available 24/7, especially from a welfare point of view. It’s like, ‘why didn’t you respond to my email at 9 o’clock last night? I’m waiting’, and it’s like 9 am the next day. Or they’d want to know at 11 o’clock why I haven’t responded, and then they go complain to the principal. So this availability of teachers that is now expected online is huge. […] Patience and respect is really lacking.

Having to be available ‘24/7’ contributed to wider complaints about asymmetries of care and participants called for principals to better manage parents’ boundary crossing. Better support from the Department of Education and school principals was a more common request among participants than better remuneration.

## Discussion

Pandemic planning is the business of public health. Planning well, minimising burdens and making sure people are adequately cared for are the business of public health ethics. In order to prepare well, we need to document the ways that gaps in care were papered over during the COVID-19 pandemic so that they are included in plans for future pandemics.

This paper describes teachers’ provision of care to accommodate both identified needs and unsolicited requests during periods of COVID-19 restrictions. We were moved to analyse through the lens of an ethics of care because the participants described what they gave and what (if anything) they received that way. What does care ethics say about the scenarios we described, where care is both initiated by and requested of teachers in the pursuit of social goods that are not education? Current teacher-focused care ethics theories of what it was for teachers to provide care during the pandemic do not account for our findings because care was (i) concerned with social goods other than education—e.g. safety, mental health support and adequate food—and (ii) occurring outside of the parameters of the classroom. Many of the observations about tinkering in the literature in the context of physical care (e.g. aged care, disability care) are reflected in the accounts we heard of remote teaching during the pandemic. Care involved context-specific problem-solving and it involved prioritising those goods that are not usually or explicitly the central concern of schools. That is, they reported ways they were able to and felt obliged to practice care under extraordinary circumstances designed to *prevent* unnecessary human contact. An important additional finding from our study was the complication of having to perform care when it was an unwanted, unacknowledged *obligation*, rather than a choice or an explicit part of a role.

These two contexts of care work—offer and obligation—differ with respect to agency and power. In the first context, care is initiated through an active choice, often motivated by a sense of moral responsibility and the relative privilege workers hold in relation to those they assist. The second is more complex. From a care ethics perspective, this dynamic is not incidental but structurally produced. As Tronto argues, democratic societies routinely offload care responsibilities onto marginalised groups, while insulating dominant groups from dependence (Tronto, 1993). Teachers are not marginalised in the usual sense, but the work of teaching is feminised and associated with emotional labour (Isenbarger and Zembylas, 2006) and thus devalued. A large survey of Australian teachers found that 71 per cent said they are not respected. This is in contrast with responses from the public about whether they respect teachers: only 18 per cent said they did not (Heffernan *et al.*, 2019). That a large majority *feel* disrespected, or uncared for, is relevant. In feeling obligated to provide care that is outside of the goods they are explicitly employed to provide, care becomes both a site of injustice and a means of its reproduction. Teachers share in the devaluation of care: their care practices were rendered essential, yet institutionally unsupported, and politically invisible.

Do essential workers whose job does not explicitly involve community care have a moral obligation to provide it? Noddings argues that we all do, that practising care is a characteristic of the ethical self (Noddings, 2013). On the other hand, in the case of workers, a requirement to provide care outside of what is specified by their employment—even to members of the community of which the worker is a part—is arguably the kind of subjugation that feminist ethicists have highlighted and warned against (Scully, 2023). Where care is systematically required of the same (types of) people, it is important to look to context and the social and political dynamics that create unwanted patterns of practice. Care is undoubtedly a moral and communal good, but an obligation to perform it must not disproportionately fall to feminised professions.

Relationality and reciprocity are key components of the ethics of care (Mol, 2008; Noddings, 2013). Participants in our study described an asymmetry of care that made them feel resentful and, in some cases, aggrieved. But the asymmetries were not individual in nature. Rather, the participants in this study felt exploited by the systems they operated within, and in which they felt compelled to compensate for institutional shortcomings. This involved supporting people situationally more disadvantaged than themselves. However, the teachers we interviewed were situationally more disadvantaged than their principals and the Department of Education and, almost without exception, said they felt let down by a lack of support from their employers. They did report a sort of reciprocity from the cared for, but were not cared for themselves.

Socially patterned fissures in care could have been anticipated before COVID-19 because they have been experienced in previous public health emergencies and often emerge in everyday life. Similarly, enough is known about the burden of care for it to have been predictable that people in feminised jobs that involve emotional labour would be required to carry unwanted care requests in a pandemic. As discussed above, knowing and being known in the community apparently influenced our participants when offering care that was not part of the job. This care, while perceived as necessary in the extraordinary context of the pandemic, may ordinarily be considered optional. This is an example of the extra, unmeasurable, positive valence that institutions such as schools have in the community, particularly where that community is close knit.

Molterer *et al*. focus on the push and pull of different goods that shapes the practice of care and the tinkering that occurs when goods clash and do not easily mesh (Molterer *et al.*, 2020). Care that teachers provided during the COVID-19 pandemic was necessitated because of public health restrictions that required isolation. The good of public health as it pertained to disease prevention was not commensurate with those other goods—also public health related—that require social connection for their fulfilment. Applied bioethics has seen a shift away from a dogged focus on the importance of individuals and their rights, to embrace the role of relationships and context, in thinking about how we ought to live. In an argument for taking communal goods seriously in bioethics, Heather Widdows and Sean Cordell wrote that ‘if there are communal goods that are not respected merely by respecting individual rights then recognizing and respecting communities and their goods is essential to treating the people in those communities ethically’ (Widdows and Cordell, 2011). This approach to bioethics has much in common with the seeds of care ethics. Care that is generated by and expected of workers at institutions of community importance is a common good that must be taken seriously. It is a good that accrues beyond the benefit to individuals. It is not simply about a frazzled parent having the ear of their child’s teacher, but also contributes to belonging, to feeling cared for and to a feeling of reassurance that someone will (probably) step in when social care breaks down in extraordinary circumstances.

This good—community care—was capitalised upon during the COVID-19 pandemic with a common social and commercial trope of ‘we’re all in this together’. This widely circulated discourse evoked a collective ideal of shared burden and mutual support. However, this rhetoric failed to adequately acknowledge the unequal distribution of labour required to sustain care under crisis conditions. For many participants in our study, the moral appeal of this narrative created a sense of obligation to go above and beyond, but it was rarely matched by the structural support needed to make that care sustainable or equitable.

## Conclusion

The ‘tinkering’ practices of care that teachers undertook illuminate both the fragility and the resilience of our social fabric and the urgent need to revalue care as foundational to modern society. In the wake of COVID-19, care ethics has remained largely focussed on individuals in formally recognised care roles, whether paid or unpaid (Tronto and Fine, 2022). The forms of extraordinary care described by the teachers in our study are either no longer needed or sought, or they continue, but have again become invisible as we revert to liberal democracy’s business-as-usual and move away from 2020 to 2021 notions of interdependence and solidarity. When care is invisible, it is not planned for, acknowledged or supported. Ignoring it exposes persistent institutional failures to recognise and sustain the everyday labour that holds society together in times of crisis.

The newly established Australian Centre for Disease Control (CDC) has pandemic planning as part of its remit (Australian Centre for Disease Control, 2025). Teachers are a snapshot example of all of the papering over cracks that so many people, particularly those in lower paid jobs, did. As part of preparing for future infectious disease emergencies, the CDC must think qualitatively and creatively about the sorts of care needs that might be created by attending to public health goods and have appropriate solutions for meeting those needs. These solutions ought not to rely on goodwill or asymmetric care practices that are premised on established patterns of how *ad hoc* solutions to social burdens are borne. Rather, pandemic plans should explicitly value the unacknowledged care that props up systems during times of particular need and beyond.
